# ARB might be superior to ACEI for treatment of hypertensive COVID‐19 patients

**DOI:** 10.1111/jcmm.17051

**Published:** 2021-11-11

**Authors:** Hong‐Jin Zhao, Yan Li, De‐Yu Wang, Hai‐Tao Yuan

**Affiliations:** ^1^ Department of Cardiology Shandong Provincial Hospital affiliated to Shandong First Medical University Ji'nan China; ^2^ Department of Cardiology Shandong Provincial Hospital affiliated to Shandong University Ji'nan China; ^3^ School of Medicine, Cheeloo College of Medicine Shandong University Ji'nan China

**Keywords:** ACEI, ARB, COVID‐19, hypertension

## Abstract

The administration of ACEI/ARB (angiotensin‐converting enzyme inhibitors/Angiotension II receptor blockers) in COVID‐19 (coronavirus disease 2019) patients with hypertension exhibits a lower risk of mortality compared with ACEI/ARB non‐users. In this context, an important question arises: is ACEI or ARB more suitable for the treatment of hypertensive COVID‐19 patients? Taken into consideration the following four rationales, ARB may offer a more significant benefit than ACEI for the short‐term treatment of hypertensive COVID‐19 patients: 1. ACEI has no inhibition on non‐ACE‐mediated Ang II production under infection conditions, whereas ARB can function properly regardless of how Ang II is produced; 2. ACEI‐induced bradykinin accumulation may instigate severe ARDS while ARB has no effects on kinin metabolism; 3. ARB alleviates viscous sputa production and inflammatory reaction significantly in contrast to ACEI; 4. ARB may attenuate the lung fibrosis induced by mechanical ventilation in severe patients and improve their prognosis significantly compared with ACEI. To examine the advantages of ARB over ACEI on hypertensive COVID‐19 patients, retrospective case‐control studies comparing the clinical outcomes for COVID‐19 patients receiving ARB or ACEI treatment is strikingly needed in order to provide guidance for the clinical application.

## CONFLICTS OF INTEREST

The authors confirm that there are no conflicts of interest.

## AUTHOR CONTRIBUTIONS

**Hongjin Zhao:** Conceptualization (equal); Methodology (equal); Project administration (equal). **Yan Li:** Conceptualization (equal); Project administration (equal). **De‐Yu Wang:** Investigation (equal); Writing‐original draft (equal). **Hai‐Tao Yuan:** Conceptualization (equal); Project administration (equal).

Dear Editor

The COVID‐19 (coronavirus disease 2019) outbreak caused by SARS‐CoV‐2 (severe acute respiratory coronavirus 2) is currently still threatening human health globally. The leading causes of death for COVID‐19 patients are severe ARDS (acute respiratory distress syndrome) and multiple organ failure due to systematic inflammatory responses. Notably, the clinical data published to date indicate that the most common comorbidity of COVID‐19 is hypertension (31.2%), and this is even higher (58.3%) in severe COVID‐19 patients receiving intensive care.^1^ It is therefore clear that optimization of proper treatment regimens for hypertension combined with COVID‐19 will benefit a very large number of people around the world.

As the anti‐hypertensive medicine, ACEI/ARB (angiotensin‐converting enzyme inhibitors/angiotensin II receptor blockers) were once thought to increase the mortality of COVID‐19 because of their inducible effect on the expression of ACE2 (angiotensin‐converting enzyme 2)—the known functional receptor utilized by SARS‐CoV‐2 to invade human cells for replication.^2^ However, clinical researches and a meta‐analysis have demonstrated that the administration of ACEI/ARB in COVID‐19 patients with hypertension exhibits a lower risk of mortality compared with ACEI/ARB non‐users.^3^, ^4^ In fact, SARS‐CoV‐2 infection exhausts lots of ACE2 molecules, resulting in decreased ACE2 levels and increased Ang II (angiotensin II) levels, which is known as RAS (renin‐angiotensin systems) imbalance. Such imbalance was found to be associated with severe pneumonia and hypertension. ACEI/ARB can help to recover the RAS balance through inhibiting Ang II—AT1R (angiotensin II type 1 receptor) signalling while enhancing ACE2—Ang1‐7 (angiotensin1‐7)—Mas receptor signalling to prevent inflammation progression and to facilitate control of blood pressure^5^ (see Figure). In this context, an important question arises: is ACEI or ARB more suitable for the treatment of hypertensive COVID‐19 patients?

From a cardiovascular perspective, the advantage of ACEI for prevention of MI (myocardial infarction) makes it a better long‐term choice over ARB for blood pressure control.^6^ However, given that inflammation‐associated acute cardiac injury and arrhythmia are more common complications with COVID‐19 than MI,^1^, ^7^, ^8^ it is conceivable that ARB may offer greater benefit than ACEI for the short‐term treatment of hypertensive COVID‐19 patients; consider the following four rationales:

First, under infection or inflammation conditions, a large proportion of Ang II is generated from Ang I through non‐ACE pathways, including via chymase and cathepsin‐mediated catalysis.^9^ Thus, whereas ACEI should have no effect on non‐ACE‐mediated Ang II production, ARB could inhibit the pro‐inflammatory and profibrotic roles of Ang II through blocking its binding to AT1R; this would be the case regardless of how Ang II is produced.

Second, ACEI can inhibit ACE‐mediated bradykinin degradation, leading to bradykinin accumulation in the lung.^10^ In ordinary hypertension patients, this accumulation may only induce cough; however, for COVID‐19 patients already experiencing pneumonia, bradykinin accumulation may instigate severe ARDS. Supporting this line of thinking, it is notable that icatibant, an antagonist of bradykinin, has already been suggested for ARDS therapy in COVID‐19. Moreover, the fact that ARB has no known effects on kinin metabolism should not further exacerbate inflammation for hypertensive COVID‐19 patients.

**FIGURE 1:**
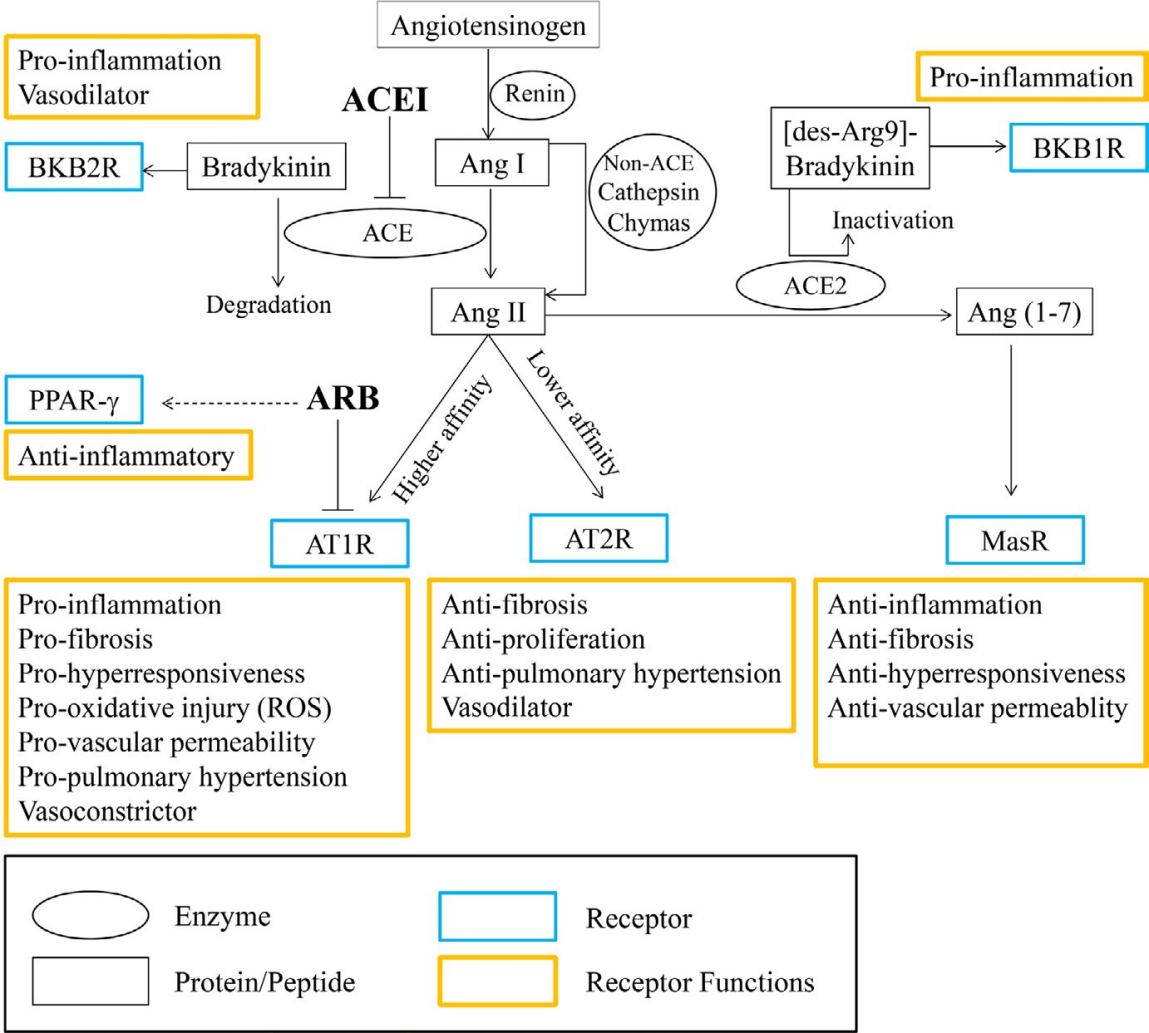
Conversion among RAS members and the correlation between RAS and other inflammation‐associated signals. Angiotensinogen is cleaved by renin to form Ang I, and the latter is further converted to Ang II by ACE, which can be inhibited by ACEI. However, in disease conditions such as infection or inflammation, the conversion from Ang I to Ang II through non‐ACE pathways (including chymase and cathepsin) is strikingly enhanced. Notably, Ang II binds to AT1R with a higher affinity to promote inflammation, fibrosis and vasoconstriction, while the binding between Ang II and AT2R is relatively lower and antagonizes the AT1R‐mediated function. Besides, Ang II can be converted by ACE2 into Ang 1–7, which stimulates the Mas receptor to counter inflammation response. Besides Ang 1–7 generation, ACE2 also mediates the inactivation of des‐Arg9‐bradykinin, or the latter is capable to stimulate BKB1R for pro‐inflammatory function. Likely, ACE promotes the degradation of bradykinin. Hence, ACEI can inhibit ACE‐mediated bradykinin degradation, resulting in the accumulation of bradykinin which can bind to BKB2R to strengthen inflammation status. ARB specifically blocks AT1R to inhibit the Ang II‐AT1R signalling, alleviating the Ang II‐associated inflammation reaction and vasoconstriction. Moreover, some ARBs, such as telmisartan, can also exert additional anti‐inflammatory effects via activating PPAR‐γ. RAS, renin‐angiotensin systems; Ang I, angiotensin I; Ang II, angiotensin II; Ang1‐7, angiotensin 1–7; ACE2, angiotensin‐converting enzyme 2; ACEI, angiotensin‐converting enzyme inhibitors; ARB, angiotensin receptor blockers; AT1R, angiotensin II type 1 receptor; AT2R, angiotensin II type 2 receptor; MasR, Mas receptor; BKB1R, bradyKinin B1 receptor; BKB2R, bradyKinin B2 receptor; PPAR‐γ, peroxisome proliferator‐activated receptor‐γ

Third, post‐mortem biopsies of COVID‐19 patients have revealed that high levels of viscous sputa are lodged in bronchia and alveoli. These sputa are not easily evacuated via coughing, and severely limit respiratory functions, likely accounting for a large proportion of deaths. Intriguingly, in ALI (acute lung injury) rat models, ARB significantly decreased the protein secretion, while ACEI had no such effect,^11^ findings suggesting a potential contribution from ARB in alleviating the production of viscous sputa. Additionally, ARB reduces inflammatory cytokines to a greater extent than does ACEI,^11^ potentially owing to ARB's blockade of AT1R. This is thought to drive increased binding of Ang II to AT2R (angiotensin II type 2 receptor) and increased conversion of Ang II to Ang 1–7 (see Figure 1), thereby attenuating inflammatory reactions synergistically with other anti‐inflammatory mechanisms of ARB mentioned beforehand.^9^ Consistently, the ARB agent irbesartan has already been shown to protect rat models from ALI via elevation of Ang 1–7‐induced signalling.^12^

Last but not least, the signalling mediated by AT2R and Mas receptor exerts anti‐fibrotic effect besides anti‐inflammatory reaction (see figure). Through AT1R blockade, ARB drives more activation of AT2R and Mas receptor compared with ACEI, which may attenuate the lung fibrosis induced by mechanic ventilation in severe patients and improve their prognosis significantly.^13^ Apart from above anti‐inflammatory mechanisms, some ARBs such as telmisartan can inhibit lymphocyte migration through activating PPAR‐γ (peroxisome proliferator‐activated receptor‐γ),^14^ which further alleviate systemic inflammatory state in hypertensive COVID‐19 patients.

Through literature review, ARB seemed to be used more often than ACEI during the pandemic period, though different medical centres showed different preference.^15^ To provide guidance for the application of ACEI/ARB in hypertensive COVID‐19 patients, we suggest a pressing need for retrospective case‐control studies comparing the clinical outcomes for COVID‐19 patients who have received ARB or ACEI treatment. There are likely large populations of such patients, and their data should be examined prior to commencing any perspective randomized controlled trials.

## Data Availability

The authors confirm that citations for available data have been included in references section.
